# Multi-wall carbon Nanotube surface-based functional nanoparticles for stimuli-responsive dual pharmaceutical compound delivery

**DOI:** 10.1038/s41598-024-59745-6

**Published:** 2024-05-27

**Authors:** Masoumeh Nabitabar, Maryam Shaterian, Hossein Danafar, Morteza Enhessari

**Affiliations:** 1 Chemistry Department, Faculty of Science, Zanjan University, Zanjan, Iran; 2 Zanjan Pharmaceutical Nanotechnology Research Center, Zanjan University of Medical Sciences, Zanjan, Iran; 3 Fachbereich Biologie, Chemie, Pharmazie, Institut für Chemie und Biochemie—Anorganische Chemie, Freie Universität Berlin, Fabeckstr, Germany

**Keywords:** Drug delivery, Breast cancer, ƒ-MWCNT, Load-release, MTT assay, Cancer, Cell biology, Chemical biology, Chemistry

## Abstract

Carbon nanotubes (CNTs) have the potential to serve as delivery systems for medicinal substances and gene treatments, particularly in cancer treatment. Co-delivery of curcumin (CUR) and Methotrexate (MTX) has shown promise in cancer treatment, as it uses fewer drugs and has fewer side effects. This study used MTX-conjugated albumin (BSA)-based nanoparticles (BSA-MTX) to enhance and assess the efficiency of CUR. In-vitro cytotoxicity tests, DLS, TEM, FTIR, UV/Vis, SEM, and DSC studies assessed the formulations' physical and chemical properties. The Proteinase K enzyme was used to severe amidic linkages between MTX and BSA. The findings demonstrated the efficacy of using ƒ-MWCNT-CUR-BSA-MTX as a vehicle for efficient co-delivery of CUR and MTX in cancer treatment. The MTT colorimetric method was used to evaluate the effect of chemical and medicinal compounds. Cell division was studied using the MTT method to investigate the effect of pure MWCNT, pure CUR, MTX-BSA, and ƒ-MWCNT-CUR-MTX-BSA. Studies on cell lines have shown that the combination of curcumin and MTX with CNT can increase and improve the effectiveness of both drugs against cancer. A combination of drugs curcumin and methotrexate simultaneously had a synergistic effect on MCF-7 cells, which indicated that these drugs could potentially be used as a strategy for both prevention and treatment of breast cancer. Also, ƒ-MWCNT-CUR-MTX-BSA was found to have a significant effect on cancer treatment with minimal toxicity compared to pure curcumin, pure MTX-BSA, MTX, and ƒ-MWCNT alone. Unique properties such as a high ratio of specific surface area to volume, high chemical stability, chemical adsorption ability, high capacity of drug and biomolecules of carbon nanotubes, as well as multiple drug loading at the same time The combination of ƒ-MWCNT-CUR-BSA MTX significantly impacts cancer therapy), are desirable as an alternative option for targeted drug delivery and high therapeutic efficiency.

## Introduction

Cancer is a collection of diseases that involve uncontrollable growth and spread of cells from the initial site to other parts of the body. Recent statistics from the World Health Organization indicate that cancer has emerged as a major cause of death, with nearly 13% of all fatalities attributed to this disease. Cancer treatment often involves the use of radiation therapy, chemotherapy, and surgery. A significant challenge associated with chemotherapy is the distribution of the drug throughout the body, which can potentially harm healthy cells^1–4^. Chemotherapy poses a major challenge in terms of ensuring the drug is distributed evenly throughout the body without causing harm to healthy cells^5^. In the past decade, there has been a growing focus on producing and advancing nanoparticles for various fields because of their unique physical and chemical properties^6–9^. Lately, there has been a significant focus on them, especially in the biomedical field, as they have the potential to be a revolutionary tool for developing drug delivery systems. Solid, almost spherical particles with a size range of 1–100 nm, called nanoparticles, can be produced either chemically or spontaneously^10,11^. Among these, nanocrystalline semiconductor particles are considered for drug delivery due to their unique characteristics such as nontoxicity, high activity, strong magnetic conductivity, solubility, and high surface-to-volume ratio. These particles are used as nanoparticles in various medical and therapeutic fields^12,13^. Carbon is a widely occurring mineral on Earth. It can be found in the atmosphere as carbon dioxide and is a natural component of many living materials. In nature, pure carbon exists in the crystal structures of graphite, diamond, lonsdaleite, and chaoite^14,15^. Carbon nanotubes (CNTs) are one of the carbon allotropes that can have an open or closed end. The number of layers of rolling graphene can distinguish between single-wall carbon nanotubes (SWCNTs) that have an outer diameter of 0.8–2 nm and multi-wall carbon nanotubes (MWCNTS) that have an outer diameter of 5–20 nm. Size can vary from several centimeters to as small as 100 nm^16–19^. CNTs are also designed to assist with distributing or targeting drugs. Studies have been conducted on their therapeutic potential, especially in treating cancer and developing novel diagnostics and nanosensors. It is expected that they will facilitate the integration of molecular imaging in diagnosing and treating illnesses, particularly in the field of oncology. Researchers are searching for ways to reduce the toxicity of these materials for use in biological applications, as their toxicity is a major concern. Surface changes, specific particle size ranges, and other elements played a role in addressing this concern^20–26^. In evaluating CNT applications for lung cancer diagnosis and treatment, Sheikhpour and colleagues emphasized the importance of surface functionalizing CNTs to the appropriate dimensions. By increasing the length, width, and curvature, some of the negative side effects such as inflammation, fibrosis, and carcinogenesis can be partially reduced^27,28^. It is important to note the significance of cancer inhibitors and their undeniable efficacy in therapy. MTX is a drug that's commonly used in cancer treatment. It works by competitively inhibiting the DHFR enzyme, which in turn suppresses the production of thymidine. MTX specifically affects the process of mitotic cell division. It is important to note that the teratogenic effects of MTX vary depending on the dosage and duration of use. Despite their lower efficacy, traditional dosage forms remain a useful approach for achieving optimal therapeutic benefits with fewer adverse effects^29–31^. Although they may not be as effective, traditional dosage forms are still a valuable method for obtaining maximum therapeutic benefits while minimizing negative side effects^32,33^.

The plant known as Curcuma longa is responsible for producing turmeric, which contains a substance called curcumin (CUR). Through human clinical trials, CUR is an affordable and safe option, with a diverse range of biological properties that make it effective for treating many different illnesses^34^. Although CUR has many positive traits, its ability to be absorbed by the body and its usefulness as therapy are limited due to its extremely low solubility in water-based solutions. Two separate trials evaluated the effectiveness of combining CUR and gemcitabine in treating advanced pancreatic cancer^35,36^. According to Kanai et al., curcumin was safe at 8 g per day along with gemcitabine and was well tolerated and safely tolerated. Epelbaum and colleagues discovered that some patients at this dosage complained of stomach aches and did not respond especially well to treatment. Patients with advanced and metastatic breast cancer who were also taking docetaxel recently participated in Phase 1 research on CUR^37,38^. Recently, many multifunctional delivery techniques have been developed for administering various pharmacological drugs. Due to their unique properties, biological carriers like bovine serum albumin (BSA), which are utilized as drug delivery vehicles, have attracted considerable interest^39^. In another study, BSA was selected from several carriers to provide MTX and CUR simultaneously. BSA offers various advantages as a macromolecular carrier for MTX and CUR, including biodegradability, circulatory stability, no immunogenicity or toxicity, a long half-life, straightforward blood flow to all tumor regions, circulatory stability, high chemical stability, and ease of administration^40,41^.

In this study, to efficiently deliver methotrexate (MTX) and curcumin (CUR) to cancer cells, a dual drug delivery system was developed using multiwall carbon nanotubes (MWCNTs).

The BSA-MTX was produced by the covalent connection of MTX and BSA. CUR and MTX-BSA were physically loaded onto the ƒ-MWCNTs by physical encapsulation, and their loading capacity and drug release profile were evaluated. The in vitro anticancer effects of these nanoparticles on the MCF-7 breast cancer cell line were investigated in the study using the MTT test. The results of this work provide major insights into the development of albumin conjugates and ƒ-MWCNT-based drug delivery systems, which show great potential for the treatment of certain cancers.

## Experimental

### Materials

The necessary chemicals were obtained from various sources. Bovine Serum Albomine (BSA), Curcumin (CUR), N-(3-Dimethylaminopropyl)-N0-ethyl Carbodiimide hydrochloride (EDC), and N-Hydroxy Succinimide (NHS), as well as 3-(4, 5-dimethylthiazol-2-yl)-2, 5-diphenyl tetrazolium bromide (MTT), were acquired from Sigma Aldrich Chemicals in St. Louis, MO, USA. Methotrexate sodium (MTX) was purchased from Zahravi Company, and a Multi-walled carbon nanotube (MWCNT) was obtained from Nano Gostar Sepa, both in Iran. All other necessary solvents were purchased from Emertat Chimi Company in Tehran, Iran. The report of the prepared ingredients and their purity are available in Table 1.

**Table 1:** Descriptions of the used chemicals, supplier CAS number, purity, and chemical structure of the material.

Chemical name	Provenance	CAS No	Mass fraction (^a^purity)	Structure
Multi-walled nanotube carbon	Sigma Aldrich	308,068–56-6	> 0.95	
Methotrexate	Merck	34,157–83-0	> 0.98	
Curcumin	Sigma Aldrich	458–37-7	> 0.94	
Bovine Serum albumin	Sigma Aldrich	9048–46-8	> 0.98	
N-(3-Dimethylaminopropyl)-N0-ethyl-Carbodiimide hydrochloride (EDC)	Sigma Aldrich	25,952–53-8	> 0.98	
N-Hydroxy Succinimide (NHS)	Sigma Aldrich	6066–82-6	> 0.98	
Proteinase K Enzyme	Sigma Aldrich	39,450–01-6	-	
DMSO	Sigma Aldrich	67–68-5	≥ 0.999	

^a^The purities were provided by the suppliers.

### Cutting and oxidation of MWCNTs

MWCNT (1 g) is prepared by suspending it in piranha solution, a combination of sulfuric acid and hydrogen peroxide in a 4/1 ratio, and stirring it for 5 h at room temperature. Then it is diluted with 1L of distilled water, agitated for a short period, and filtered through 0.22 m filter paper. After being separated in the filter, the MWCNTs are cleaned with distilled water until the pH of the filtrate is neutral, then with a 0.01 M sodium hydroxide solution and distilled water again until the pH is neutral. Finally, it is washed until the pH of the filtrate is neutral using a 0.01 M hydrochloric acid solution and distilled water. This gives MWCNT-COOH (ƒ-MWCNT) as a black solid^42^.

### Conjugation of MTX on BSA (BSA-MTX)

A compound called BSA-MTX was created by combining MTX (100 mg) and BSA (200 mg) using EDC (183 mg) and NHS (21.85 mg) in deionized water (20 ml) under basic conditions. At RT, the reaction was allowed to continue for 24 h. To isolate the BSA-MTX compound, a dialysis process with a 12 kDa membrane was employed^29^.

### Physically loading of CUR and MTX-BSA on MWCNT

A solution was prepared by dissolving 0.02 g of carbon nanotubes (CNT) and 0.1 g of methotrexate-conjugated albumin-based nanoparticles (BSA-MTX) in 8.5 ml of water. Additionally, 0.04 g of curcumin was dissolved in 1.5 ml of dimethyl sulfoxide (DMSO). The obtained solutions were combined and subjected to stirring at room temperature within the laboratory environment for 24 h. Subsequently, the resulting solution underwent centrifugation at 20,000 *rpm* to facilitate the separation of the desired product.

### Characterization

Various techniques were utilized to examine the samples, including FTIR, UV/Vis, TEM, DSC, and DLS. ƒ-MWCNT-CUR-BSA-MTX was photographed by a transmission electron microscope (TEM; Cambridge 360–1990 Stereo Scan Instrument-EDS) to study the particle size and shape. TEM can be used to study the growth of layers, their composition, and defects in semiconductors. High resolution can be used to analyze the quality, shape, size, and density of quantum wells, wires, and dots. The TEM operates on the same basic principles as the light microscope but uses electrons instead of light.

The FTIR spectrophotometer (Bruker, Tensor 27) was used to measure FTIR spectra. IR technique is used to determine functional groups in molecules. IR spectroscopy measures the vibrations of atoms, and based on this, functional groups can be determined. In general, stronger bonds and lighter atoms vibrate at higher stretching frequencies (wave numbers).

UV–Vis can be used to measure concentrations, identify unknown compounds, and provide information about the physical and electronic structure of organic and inorganic compounds. It is widely used in chemical, physical, and biological assays.

Additionally, thermal analysis of the NPs was conducted using (Mettler Toledo, Star SW 9.30 model, Schwerzenbach, Switzerland) DSC with heating samples at a rate of 15 °C min^−1^. DSC is used to measure enthalpy changes due to changes in the physical and chemical properties of a material as a function of temperature or time. The method allows you to identify and characterize materials. Differential scanning calorimetry is fast, very sensitive, and easy to use.

A nano/zeta sizer (Malvern Instruments, Worcestershire, UK, model Nano ZS) was used to measure the ζ-potential and hydrodynamic sizes. To determine the components of CNT, CUR, MTX, and BSA in the final formulation ƒ-MWCNT-CUR-BSA-MTX, using a UV/Vis spectrophotometer (Thermo Fisher Scientific, USA, Madison, model GENESYS-TM 10S), the samples' absorption spectra were recorded. DLS can determine the hydrodynamic size of protein monomers, small grains in the nanometer range, and to some extent particles in the upper nanometer/lower micrometer range. This technique can also be used to analyze colloidal systems such as liposomes, nanoparticles, polymers, and virus-like particles.

A scanning electron microscope, or SEM, produces detailed, magnified images of an object by scanning its surface to create a high-resolution image. SEM does this using a focused beam of electrons. The resulting images show information about what the object is made of and its physical characteristics.

### Cell culture

The Pastor Institute of Iran donated the human breast cancer cell line MCF-7 to the Michigan Cancer Foundation. RPMI medium was used to cultivate these cell lines, which contained 10% fetal bovine serum (FBS) and FBS-containing culture medium. The cell cultures were kept in an incubator at a temperature of 5% carbon dioxide in a humid atmosphere at a temperature of 37 °C. The Nikon Eclipse 80i inverted microscope, produced in Tokyo, Japan, was used to evaluate cell morphology.

### MTT assay

To evaluate the impact of chemical and pharmaceutical compounds, the colorimetric MTT assay was utilized. MCF-7 cells were originally sown in 96-well plates and left to settle. Subsequently, the cells were exposed to six different concentrations of the studied compounds, ranging from 75 to 300 µl ml^−1^, in RPMI medium, which were diluted, using a 0.22 m syringe filter, which had been sterilized. Sigma-Aldrich's MTT solution, which contains 3 (4, 5-dimethylthiazol-2-yl)-2, 5-diphenyltetrazolium bromide (MTT), was applied to each well after 24 h. After a 4-h incubation period, to dissolve the formazan crystals, substitute the medium with 150µL of dimethyl sulfoxide (DMSO).

The dish was shaken for about an hour while kept away from direct sunlight. The optical density of decreased MTT at 570–630 nm was measured with the use of a microplate reader (Synergy HT from BioTeK Instruments Inc., Winooski, VT, USA) to assess cellular viability a ratio between control cells and the absorbance of treated calculated using the percentage of viable cells. Additionally, to find the chemical concentration that results in a 50% decrease in cell viability, IC50 was calculated using a non-linear regression, logistic equation^43,44^.

### Drug release study

The mode of release of MTX was evaluated. For this, ƒ-MWCNT-CUR-MTX-BSA was used, which was heated to 37 °C while it was in PBS at pH = 7.4 inside a dialysis bag with a cutoff for a molar weight of 12,000 Da. Then the bag was placed in 25 ml of PBS and 2 ml of the external solution was removed at time intervals. It was examined with a spectrophotometer at a wavelength of 304 nm and returned to the environment to keep the drug release test volume constant. The procedure was repeated in the presence of Proteinase K enzyme to cleave the amide bond between MTX and BSA, as well as the amide bonds within the BSA structure. To evaluate the release pattern of CUR, we incubated MWCNT-CUR-MTX-BSA in PBS with 2% (v/v) Tween 80 at pH 7.4 and pH 4.4 at 37 °C in a dialysis bag with a molecular weight cutoff of 12,000. Subsequently, this bag was immersed in 25 ml of PBS.

## Results and discussion

A CNT nanohybrid's biological performance is only dependent on its chemical-physical characteristics, which in turn are influenced by its two inorganic and organic counterparts^45,46^. This opens up an extensive array of options for the development of tailored materials. Therefore, the primary hurdle to be overcome when constructing the selection of carbon nanostructures with high-performing carrier systems and acceptable characteristics^47,48^. It is well known that the CNT features are largely determined by the synthetic and purification processes, using the ability to modify patterns of morphology (for example, the number and size of shells), the electric and magnetic responses, branch density, surface chemical affinity, and the number of defects^49,50^. In the synthetic approach, the MWCNT was first oxidized using an H_2_SO_4_/hydrogen peroxide solution to get rid of any remaining catalyst and create COOH groups that could be further functionalized. We loaded two medicines onto the carbon of the activated nanotubes (Carboxylic) concurrently using a physical approach. Combination therapy using anti-cancer medications and genes has recently emerged as a new cancer treatment strategy. Drug resistance and toxicity are both reduced as a result of this strategy's synergistic effects. Numerous researchers noted such synergies. In this work, BSA molecules were employed as a smart and biocompatible delivery mechanism for combination treatment. More precisely, the carboxyl groups of MTX were activated using EDC/NHS and subsequently reacted with BSA to form a potent smart biomacromolecule carrier that is especially targeted to tumors. The final formulation was created when the MTX-BSA and CUR molecules were physically loaded through hydrogen bonding and interaction. Through the endocytosis route, When MTX interacts with its receptor on the cell surface, it can trigger cellular internalization. In several cancer types, including brain, lung, ovarian, breast, and cervical cancers, the folate receptor is activated. However, normal cells only express a small amount of folate receptors. On many different cancer types, MTX has a therapeutic impact in addition to a targeted role.

In light of this, our team proposed that MTX alteration may provide BSA with a more precise tumor-targeting property. Since covalent bonds are relatively robust and are thus most likely to be destroyed only in lysosomes' severe conditions, the covalent fusion of medicines on carriers is preferred^29^.

### Synthesis of ƒ-MWCNT-CUR-BSA-MTX

Albumin has been widely utilized as a biocompatible delivery vehicle due to its biodegradability, stability in extended half-life, and blood circulation. The clinical success of Abraxane^®^ serves as a prime example of albumin's potential as a carrier^51^. In this study, the functional groups of albumin were employed for the conjugation of therapeutic agents (MTX) and targeting. The BSA-MTX was created by combining BSA and MTX through NHS and EDC activation, linking them at room temperature. In addition, CUR, which acts as both an anticancer agent and protector of normal cells, was physically loaded to create ƒ-MWCNT-CUR- BSA-MTX. Notably, no specific method or procedure was used in the preparation of nanoparticles. I prepared the ƒ-MWCNT-CUR-MTX-BSA using a straightforward approach that resulted in a polydispersity index (PDI) and a narrow particle size distribution of approximately 0.36.

We used ultraviolet spectrophotometry to determine the MTX content of the conjugated nanoparticles. The calibration curve for MTX concentrations was measured at 304 nm. After analyzing the data, it was found that 8.60 ± 1.63 mg MTX was linked per 100 mg BSA according to the optimal prescription. The ideal ƒ-MWCNT-CUR- BSA-MTX was found to have a mean particle size of around 140 nm, a PDI of 0.366, and a zeta potential of -23.42 mV. The loading efficiency of CUR was found to be 3.61 ± 0.16%.

### Characterization

**Figure 1:**
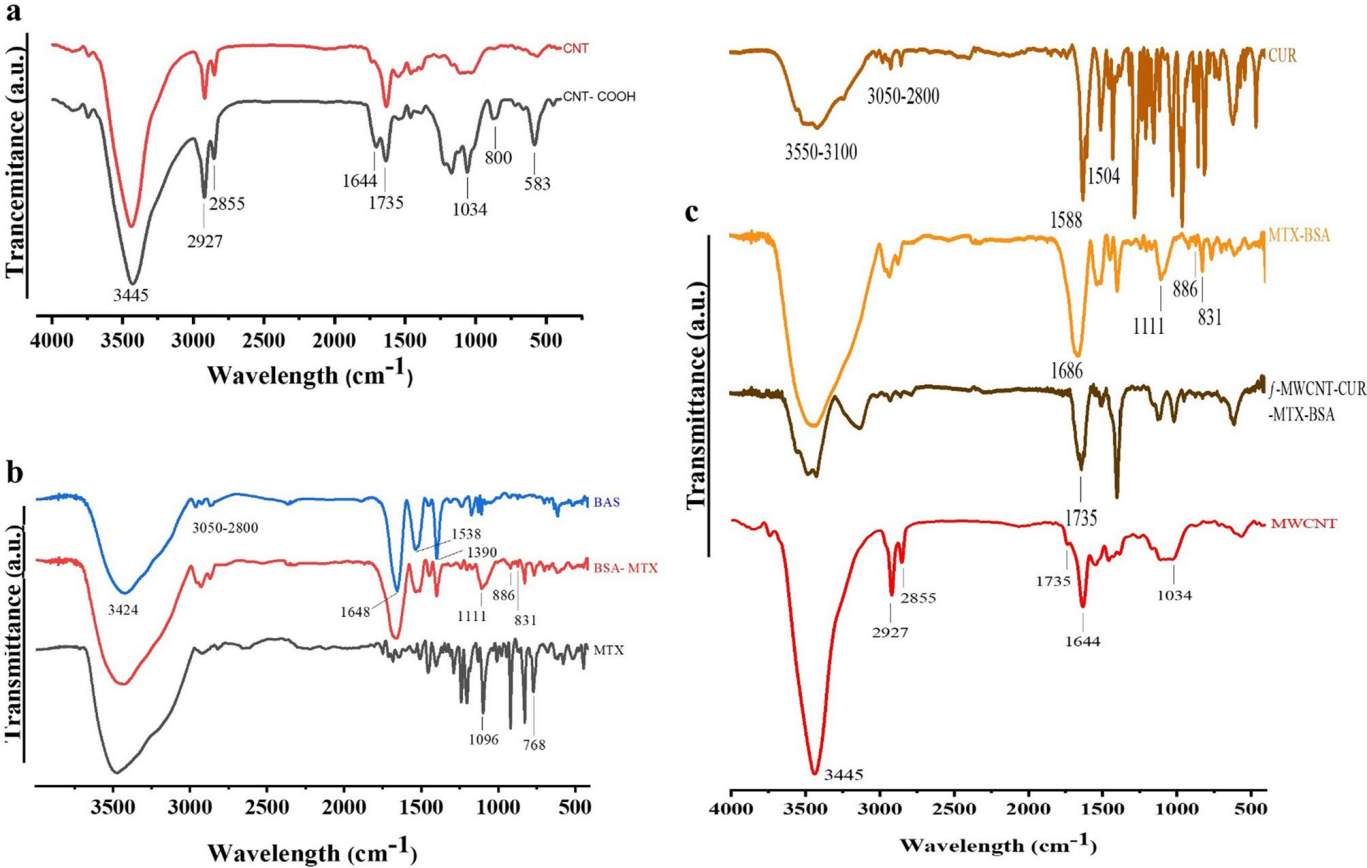
FTIR spectrum from 400 to 4000 cm^−1^ (**a**) CNT and CNT-COOH, (**b**) BSA, MTX, and MTX-BSA (**c**) ƒ-MWCNT, ƒ-MWCNT-CUR-BSA-MTX, MTX-BSA and CUR.

This study aims to analyze the composition of several compounds, such as MWCNT, MWCNT-COOH, CUR, MTX, BSA, BSA-MTX, and ƒ-MWCNT-CUR-BSA-MTX, using FTIR analysis. The purpose of the analysis is to identify the surface functional groups in each compound and track any changes that occur during chemical functionalization. Based on the results in Fig. 1a, it was found that the peak in the FTIR spectrum at 1644 cm^−1^ of MWCNTs is equivalent to the C–C stretching, which suggests the graphite structure of MWCNTs. Additionally, the emergence of a new peak at 1735 cm^−1^ was observed in the MWCNTs-COOH spectrum, which confirmed the presence of C–O stretching in the group of carboxylic acids^50–52^. The peaks of MWCNT-COOH seen at 3445 cm^−1^ and 1034 cm^−1^ correspond to the O–H and C–O stretching vibrations of the COOH group, respectively. The presence of symmetric and asymmetric C–H stretching peaks at 2855 cm^−1^ and 2927 cm^−1^, respectively, suggests that the acid treatment effectively introduced carboxylic acid groups. The appearance of strong aliphatic C–H stretching vibrations at 2891 and 2935 cm^−1^, and an amide (I) band at 1634 cm^−1^ confirmed the condensation reaction between MWCNT–COOH. Additionally, the lack of a 1680 cm^−1^ peak indicated that thioester moieties were not present in the carbon nanotubes^53,54^. Chemical functionalization allowed for the successful functionalization of MWCNTs in a two-step process. These results offer valuable knowledge regarding the compositional and structural alterations that occur during MWCNT functionalization, which may have a profound effect on the growth of innovative applications for these compounds. The peaks of BSA's absorption were analyzed and found to be 1538 and 1648 cm^−1^, which were attributable to Amide I and Amide II, respectively, flexural vibration adsorption. Upon analyzing the spectrum of the FTIR BSA-MTX, new peaks were discovered at 831, 1111, and 1687 cm^−1^, indicating that MTX was conjugated on BSA through amide bonds in Fig. 1b. pi-bonds (π-bonds) are formed by the overlap of two adjacent p-orbitals. If a p-orbital is present on an atom adjacent to the pi bond, then it can also overlap with the p-orbitals from the pi-bond, provided that it is aligned in the same plane. This overlap is called conjugation and allows for the de-localization of electrons we call resonance. Atoms containing a p-orbital include carbocations, atoms bearing lone pairs, atoms that participate in an adjacent pi-bond, and atoms bearing a free radical. Conjugation of an atom with an adjacent pi-bond will affect its bond lengths and electron distribution. Conjugation is impossible on the bridgehead atom of many bicyclic molecules since the p-orbital on the bridgehead cannot properly overlap with p-orbitals on adjacent atoms. Here, conjugation is expected between the amide nitrogens of methotrexate and BSA. The spectra of the FTIR ƒ-MWCNT-CUR- BSA-MTX exhibited each of the distinctive absorption peaks for CUR and BSA-MTX, as shown in Fig. 1c.

**Figure 2:**
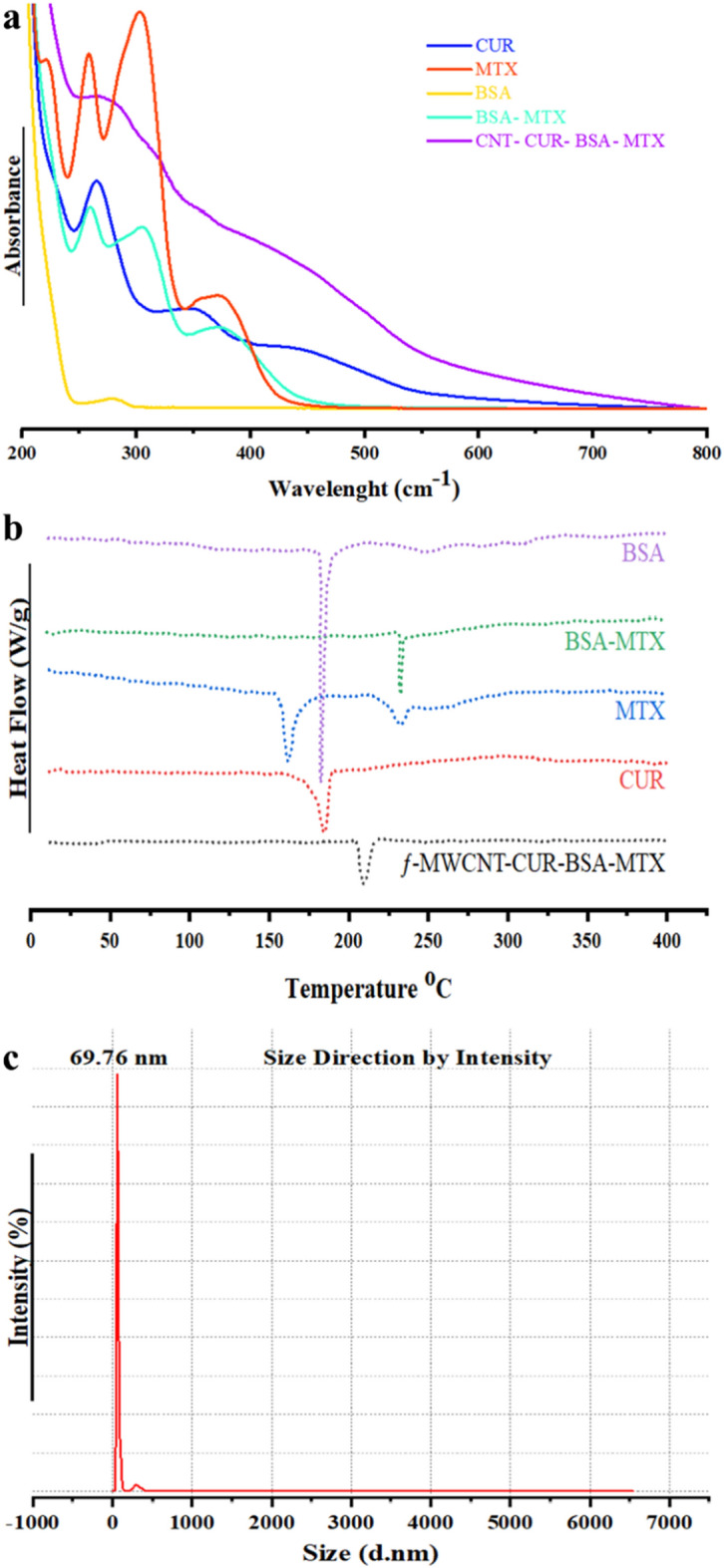
UV–Vis spectra of CUR, MTX, BSA, MTX-BSA, and CUR-MTX-BSA in PBS (**a**), DSC thermograms of CUR, MTX, BSA, MTX-BSA, and ƒ-MWCNT-CUR-MTX-BSA in the temperature range of 30–300 °C (**b**), Hydrodynamic size distribution of ƒ-MWCNT-CUR-BSA-MTX (**c**).

The optical properties of MTX conjugation, MTX-BSA, and CUR loading were assessed using an ultraviolet-violet spectrophotometer. In Fig. 2a, UV–Vis absorption at around 279 nm was used to investigate the conjugation of MTX to BSA molecules. Three distinctive absorption peaks were visible in MTX's UV–Vis absorption spectra at 260, 305, and 374 nm. Additionally, Fig. 2a displayed the range of UV–Vis absorption of BSA-MTX, where the peak at around 286 nm corresponded to BSA, while peaks at 263, 308, and 377 nm corresponded to MTX. The presence of four distinct peaks in BSA-MTX at 286, 263, 308, and 377 nm indicated successful conjugation of MTX to BSA. In Fig. 2a, the loading of CUR and BSA-MTX into the CNT carrier utilizing the UV–Vis absorption. The UV CUR's spectrum exhibited a strong absorption peak strong peak at 270 nm and a wavelength of 428 nm. Meanwhile, the ƒ-MWCNT-CUR-BSA-MTX had all the distinguishing peaks (278, 360, 437, 450, and 508 nm), indicating the successful loading of CUR onto the BSA-MTX carrier and conjugation of MTX with BSA.

DSC was also used to check the presence of drug and conjugation in the formulation. As depicted in Fig. 2b, it is evident that BSA and MTX had melting points of 171.03 °C and 154.27 °C, respectively. The DSC thermogram study of BSA-MTX shows a single endothermic peak at 214.30 °C, proving that MTX is the cd to the BSA molecules. Based on Fig. 2b, it can be observed that the CUR powders displayed a distinct and concentrated endothermic melting peak at 170.83 °C. Conversely, the BSA-MTX and CUR-loaded ƒ-MWCNT nanoparticles (ƒ-MWCNT-CUR-BSA-MTX) showcased just one endothermic peak at 182.18 °C on their DSC thermogram. It is worth noting that the CUR characteristic peak was absent in the thermogram of ƒ-MWCNT-CUR-BSA-MTX. It appears that neither CUR nor BSA-MTX were in their crystalline state after being loaded into the ƒ-MWCNT-CUR-BSA-MTX nanoparticles. The outcome showed that MTX, BSA-MTX, and CUR were successfully conjugated and loaded onto ƒ-MWCNT.

The nanoparticles of ƒ-MWCNT-CUR-BSA-MTX that have been prepared are of an appropriate size of approximately 250 nm (Fig. 2c), with a polydispersity index (PDI) of 9.95, making them suitable for use in biological applications. The nanoparticles have a polydispersity index (PDI) of 5.35, which indicates a significant level of variability in their size distribution. Particle size less than 300 nm is expected to be ideal for effective drug delivery. DLS can also be used to measure the zeta potential of nanoparticles, which is another important result. High zeta potential suspensions exhibit excellent electrostatic stability. In this study, it was observed that the ƒ-MWCNT-CUR-BSA-MTX and pure ƒ-MWCNT have a positive charge of approximately 34.1 and 30.3 mv, respectively, as depicted in Table 2. Based on the research's findings^55^, the conclusion was that the particles had a positive charge, contributing to the excellent physical stability of the formulations. Looking at Zeta potential values can provide insights into surface chemistry and the density of active functional group changes in ƒ -MWCNT treated. When the pH is increased from alkaline to acidic, the -COOH groups lose more protons, causing ζ-potential Moving to the negative side. This is a natural chemical process. The potential was attained through the ionization of fundamental groups. The amount of MWCNT in purified is lower. Purified MWCNT shows less zeta potential in compaction-loaded CUR and BSA-MTX molecules on ƒ-MWCNT given the availability of a few free carboxylic groups. These substantial changes in zeta potential point to the conjugation of ƒ-MWCNT-CUR-BSA-MTX the functionalized molecules MWCNT. The loading ƒ-MWCNT-CUR-BSA-MTX molecules on MWCNT significantly alters the zeta potential, which represents the loading of ƒ-MWCNT-CUR-BSA-MTX in nanotubes^56^.

**Table 2:** DLS and Z-potential Obtained results.

		Size (d.nm)	% intensity	St Dev (d.nm)
Z-average (d.nm): 58.1	Peak 1:	233	98.5	56.1
PDl: 9.95	Peak 2:	934	1.5	215.9
	Peak 3:	0.00	0.00	0.00
Result quality: efficient				
Zeta potential (mv): 34.1	PDl: 9.95		Conductivity (uS/cm): 48	
Result quality: efficient				

**Figure 3:**
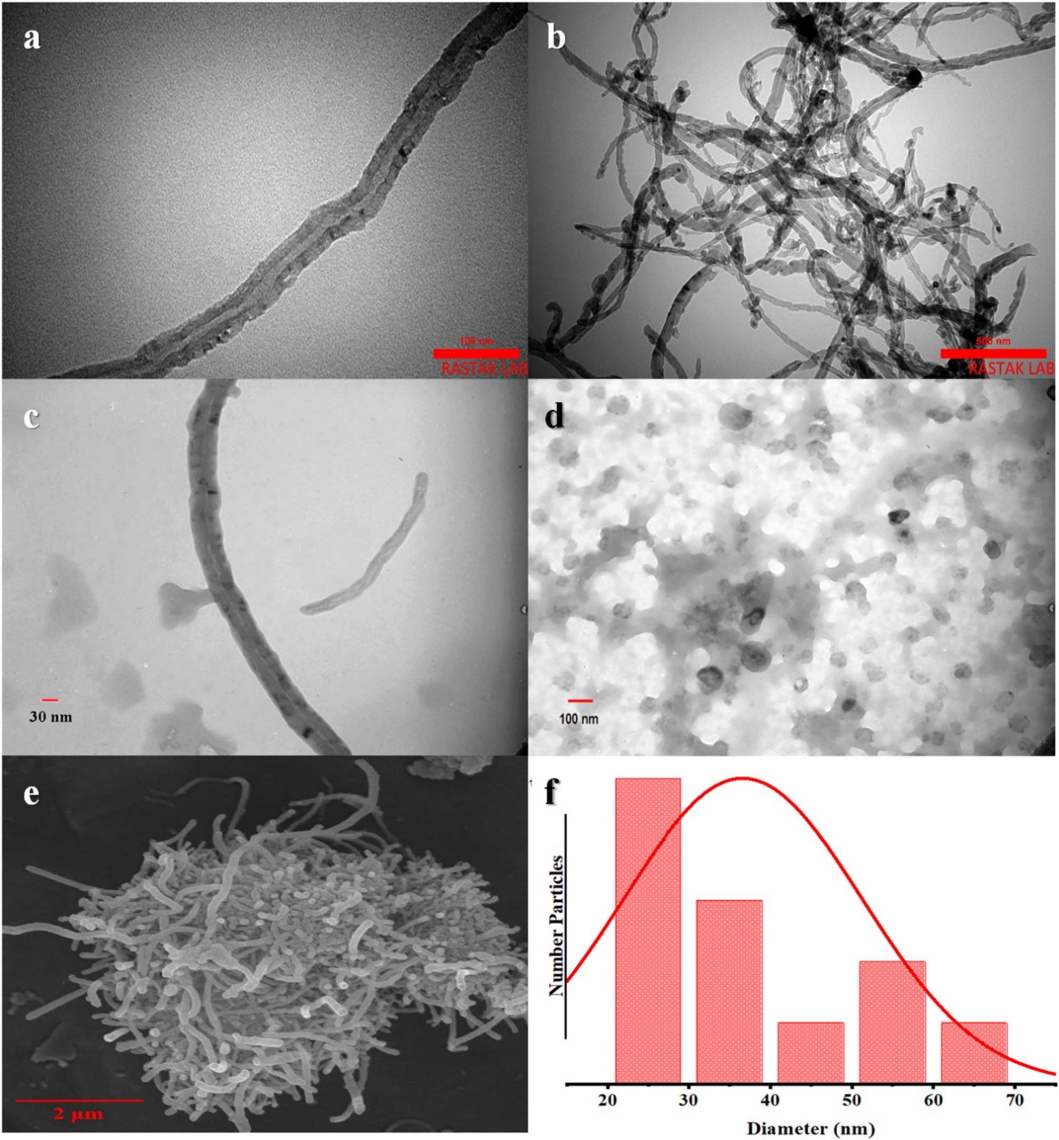
The TEMs of MWCNT (**a**), ƒ-MWCNT (**b**), ƒ-MWCNT-CUR-BSA-MTX (**c**), and BSA-MTX (**d**), The SEM of ƒ-MWCNT-CUR-BSA-MTX (**e**), and Corresponded size histogram of ƒ-MWCNT-CUR-BSA-MTX (**f**).

The topography of carbon nanotubes with and without factor group, ƒ-MWCNT-CUR-BSA-MTX and MTX-BSA was examined using electron microscopy; the findings are displayed in Fig. 3a–d, respectively. The TEM microphotographs clearly showed that the oxidized MWCNTs and ƒ-MWCNT-CUR-BSA-MTX consisted of a tubular shape structure and were in the nanometric size range. Additionally, Due to oxidation, it was shown that the size of carbon nanotubes decreased during carboxylation and that this drop was related to the time spent in oxidation. The size and shape of ƒ-MWCNT-CUR-BSA-MTX nanoparticles were also examined using scanning electron microscopy (SEM) image. Tubular nanoparticles have a consistent shape, as Fig. 3e illustrates. Their average size also closely resembles the TEM's findings. Also, Fig. 3f shows the histogram of the corresponding size of CUR and BSA-MTX loaded on the carbon nanotubes. The results showed that ƒ-MWCNT-CUR-BSA-MTX nanoparticles showed tubular morphology and uniform particles with an average diameter of 36.67 nm.

### Drug release study

**Figure 4:**
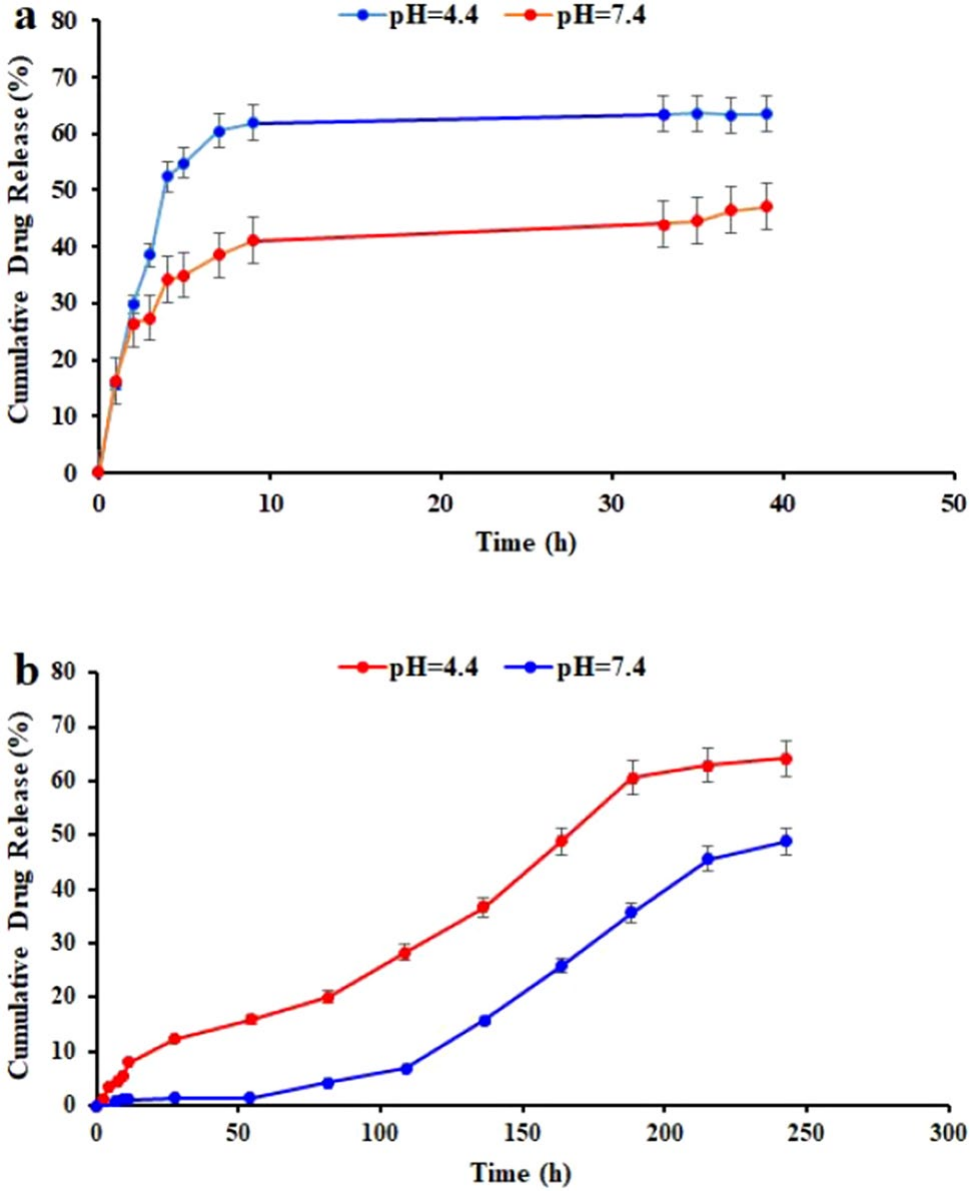
The release profiles of the MTX from ƒ-MWCNT-CUR-BSA-MTX with and without Proteinase K enzyme at pH 7.4 (**a**), the release profiles of the CUR from ƒ-MWCNT-CUR-BSA-MTX in different PH (**b**).

This study investigated the release of methotrexate (MTX) amin-modified and curcumin from CNT nanoparticles. Prior research has shown that the covalent bond between the MTX with amine-modified nanoparticles is hydrolyzable under intracellular circumstances. To evaluate the release of MTX, we conducted experiments and observed that MTX release occurred before the 12-h distance. Our data suggest that the peptide bond was easily cleaved by protease, indicating that the enzymatic MTX is released enzymatically more quickly than the hydrolytic release. Moreover, it has been tested the release of MTX in the proteinase K enzyme's presence and has been found that the drug was released faster. Approximately, the release of 67% of MTX from ƒ-MWCNT-CUR-BSA-MTX after 12 h in the presence of an enzyme, Nevertheless, just 48% Without an enzyme, MTX was released from the nanoparticles (Table 3). It is important to note that the ideal activity of the enzyme is at a pH of 7–8, and physiological PH was used to do this test. The enzymatic degradation of amidic bonds and carriers was evaluated using this test, and it is not suitable for determining pH value. This study highlights the importance of the lysosomal compartment in drug release. As evidenced by the data, the lysosomal compartment is the best place for drug release and breakdown. Overall, this study provides insights into the mechanisms of drug release from nanoparticles and the potential use of enzymes to enhance the release of drugs, Fig. 4a. As is also obvious, More CUR can undoubtedly be released with a drop in PH. While the cumulative CUR release rates of 36 and 60% at 188 h, respectively, were comparable, the nanoparticles at physiological and acidic pH displayed distinct drug release patterns. Due to the amidic bonds of the carrier breaking down in acidic media, the initially produced nanoparticles exhibited pH-sensitive drug release. At neutral pH (PH = 7.4), as shown in Fig. 4b, the cumulative release of CUR from ƒ-MWCNT-CUR-BSA-MTX was very low. Due to the acidic nature of the tumor area's microenvironment, lysosomes, and endosomes, which can help the drug release to the appropriate spot, this trait is beneficial in the delivery of anticancer drugs.

**Table 3:** The release profiles of the MTX with and without Proteinase K enzyme at pH 7.4.

X (time)	Y (without proteinase K enzyme)	Y (with proteinase K enzyme)
0	0	0
1	0.162	0.155
2	0.264	0.298
3	0.274	0.385
4	0.341	0.524
5	0.35	0.548
7	0.385	0.605
9	0.411	0.619
33	0.441	0.635
35	0.446	0.636
37	0.464	0.6324
39	0.471	0.63416

The release profiles of the CUR at pH 4.4 and 7.4		
X (time)	Y (7.4)	Y (4.4)
0	0	0
2	0.00487	1.19526
4	0.67136	3.58985
7	0.89037	4.33527
9	1.03694	5.38193
11	1.18432	8.07574
27	1.46448	12.25146
54	1.43286	15.96436
82	4.24647	20.1263
109	6.91027	28.3336
136	15.71602	36.69032
163	25.8709	48.79237
189	35.729	60.59723
215	45.8425	62.8128
243	48.84731	64.12828

### Cytotoxicity assay

**Figure 5:**
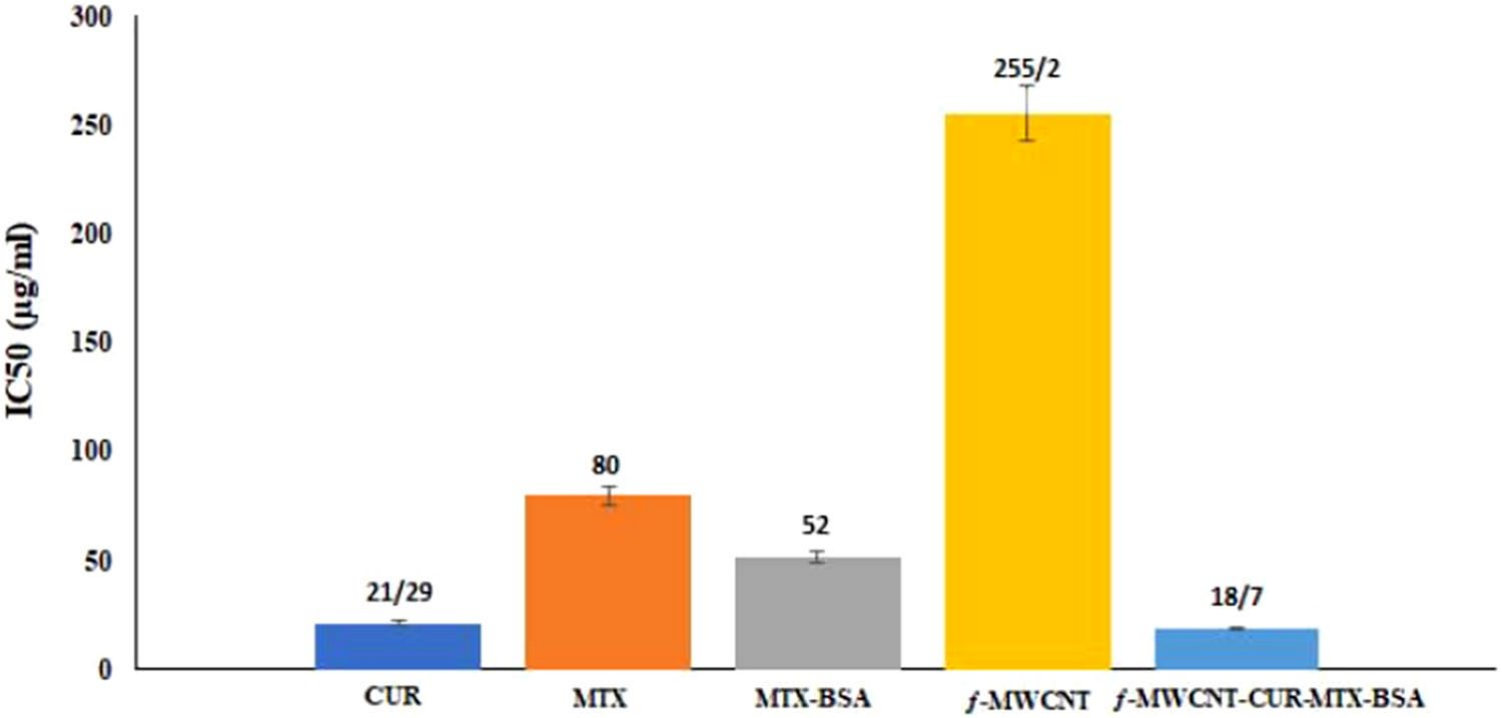
IC50 amounts for the investigated compounds in the MCF-7 cell line.

Cell division was studied using the MTT assay to examine the impact of pure MWCNT, pure CUR, MTX-BSA, and ƒ-MWCNT-CUR-MTX-BSA. The IC50 values were calculated based on the MTT data, indicating the concentration that can inhibit 50% of the total. This refers to the concentration of the compound that allows 50% of the cells to remain alive. The IC50 results are shown in Fig. 5, with the order of cell viability being ƒ-MWCNT-CUR-BSA-MTX > pure CUR > pure MTX > BSA-MTX > pure MWCNT. Researchers conducted comparative molecular docking studies of BSA-MTX and curcumin at various breast cancer protein receptors to assess the effectiveness of the drugs when used together. Studies on cell lines have revealed that combining curcumin and MTX with CNT can increase and improve the effectiveness of both drugs against cancer. The combination of medications has a synergistic impact against MCF-7 cells, indicating that these medications can potentially be used as a strategy for both preventing and treating breast cancer. After analyzing the data, it was found that ƒ-MWCNT-CUR-MTX-BSA had a more significant impact on treating cancer with minimal toxicity compared to pure curcumin, MTX-BSA, pure MTX, and ƒ-MWCNT by themselves. Further study of in vivo research is necessary to obtain greater confirmation.

## Conclusions

It is possible to attach proteins, drugs, and target ligands to bioactive and biomolecule molecules using carbon derivatives of nanotubes. Nanotubes have a unique carbon structure that can be one-dimensional or multi-dimensional, making them a suitable foundation for medical applications. By having a high specific surface area and high density, adsorption and many covalent bonds can occur. This study used MWCNTs with an H2SO4/hydrogen peroxide solution. When the MWCNTs underwent the process, their length and oxide grades decreased. This reduced toxicity effects and increased biocompatibility compared to MWCNTs without factor group. A new method for distributing MTX and CUR in living cells has been developed. This approach involves a biodegradable and stealthy nanostructure that specifically targets tumors, and it has proven to be highly effective. Our work describes a tumor-targeted nanocarrier that exhibits excellent biocompatibility and stability when used as a component of CNT. The ƒ-MWCNT-based MTX and CUR drug delivery mechanism is released within the lysosomal compartment. Additionally, because of their pH responsiveness, MTX-BSA and CUR-loaded demonstrated excellent controlled release capabilities for curcumin and Methotrexate. Using a nanosystem, this ƒ-MWCNT-CUR-BSA-MTX is a crucial strategy for efficiently delivering drugs. The results of studies on cell lines showed that a combination of CUR and BSA-MTX increased the anti-cancer effects of any medication in an additive or synergistic manner. This combination could hold promise for the treatment and chemoprevention of breast cancer due to its synergistic impact against MCF-7 cells Nevertheless, the decreased solubility and low absorption of both drugs limit their bioavailability. One successful strategy for drug delivery despite restrictions is the use of nanocarrier-based delivery systems. Studies have shown that using nanocarriers for drug delivery can offer advantages such as better pharmacokinetics of medicines, reduced drug interactions, and personalized drug release. This makes it a promising approach for advanced cancer treatments. Ongoing research is currently exploring the potential benefits of using a nanocarrier based on ƒ-MWCNT to deliver a combination of BSA-MTX and CUR medications. After evaluating the data and comparing them to pure ƒ-MWCNT, methotrexate, and Curcumin, it was discovered that ƒ-MWCNT-CUR-BSA-MTX had a significant impact on cancer treatment.

## Data Availability

The datasets utilized and/or analyzed during this study are available from the corresponding author on reasonable request.
