# Author Correction: Multi-wall carbon Nanotube surface-based functional nanoparticles for stimuli-responsive dual pharmaceutical compound delivery

**DOI:** 10.1038/s41598-024-66133-7

**Published:** 2024-07-03

**Authors:** Masoumeh Nabitabar, Maryam Shaterian, Hossein Danafar, Morteza Enhessari

**Affiliations:** 1 Department of Chemistry, Faculty of Science, University of Zanjan, Zanjan, 45371-38791 Iran; 2 Zanjan Pharmaceutical Nanotechnology Research Center, Zanjan University of Medical Sciences, Zanjan, Iran; 3 Fachbereich Biologie, Chemie, Pharmazie, Institut für Chemie und Biochemie—Anorganische Chemie, Freie Universität Berlin, Fabeckstr, Germany

Correction to: *Scientific Reports* 10.1038/s41598-024-59745-6, published online 27 May 2024

The original version of this Article contained an error in Figure 4 where in the drug release chart, “with enzymes without enzymes” was incorrectly given as "PH = 4.4 and PH = 7.4" in panel (a). The original Figure 4 and accompanying legend appear below.

**Figure 4:**
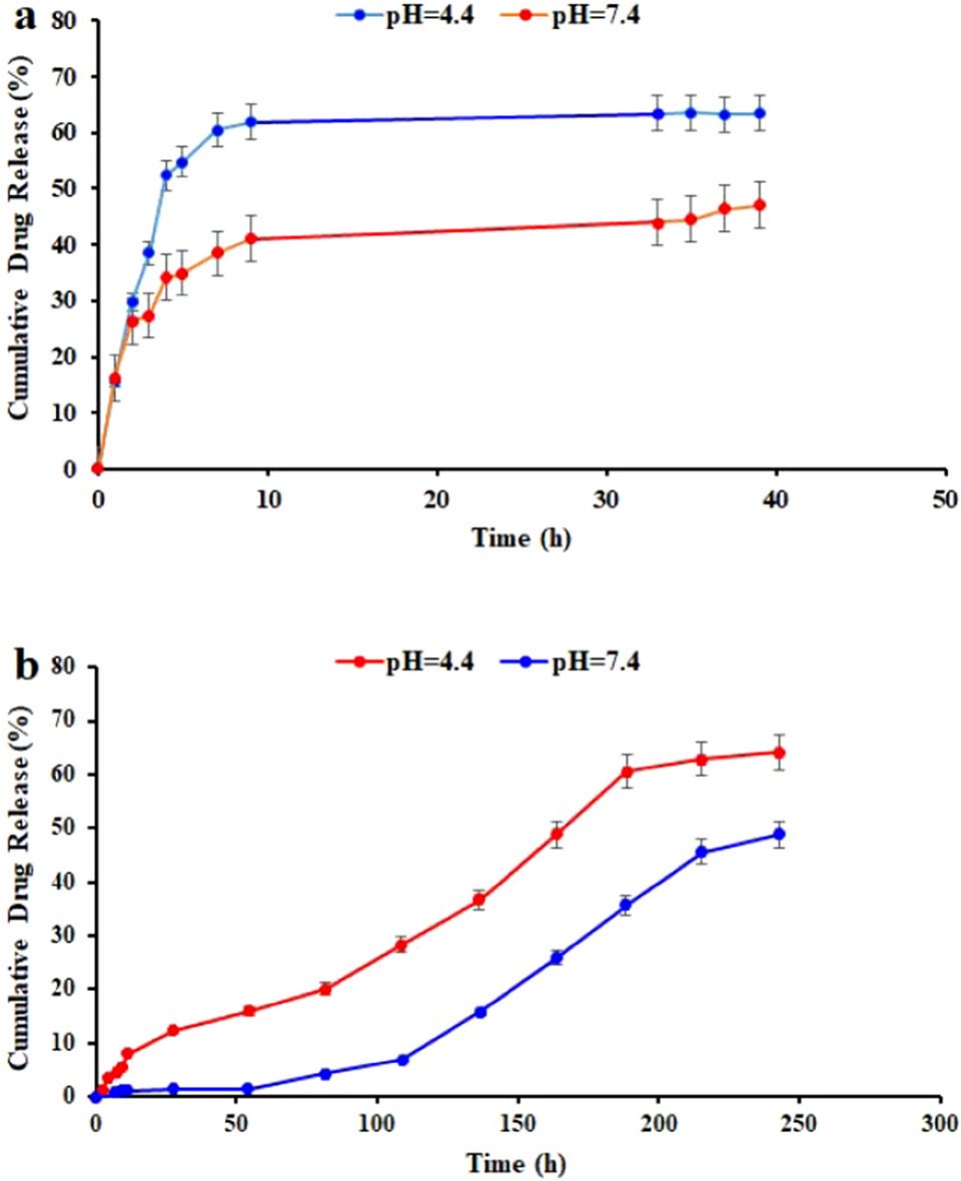
The release profiles of the MTX from ƒ-MWCNT-CUR-BSA-MTX with and without Proteinase K enzyme at pH 7.4 (**a**), the release profiles of the CUR from ƒ-MWCNT-CUR-BSA-MTX in different PH (**b**).

In addition, Affiliation 1 was incorrectly given as ‘Chemistry Department, Faculty of Science, Zanjan University, Zanjan, Iran’. The correct affiliation is listed below:

Department of Chemistry, Faculty of Science, University of Zanjan, Zanjan, 45371-38791, Iran

The original Article has been corrected.

